# An exploration of flavours in studies of e‐cigarettes for smoking cessation: secondary analyses of a systematic review with meta‐analyses

**DOI:** 10.1111/add.16091

**Published:** 2022-12-05

**Authors:** Nicola Lindson, Ailsa R. Butler, Alex Liber, David T. Levy, Phoebe Barnett, Annika Theodoulou, Caitlin Notley, Nancy A. Rigotti, Jamie Hartmann‐Boyce

**Affiliations:** ^1^ Nuffield Department of Primary Care Health Sciences University of Oxford Oxford UK; ^2^ Cancer Prevention and Control Program Georgetown University‐Lombardi Comprehensive Cancer Center Washington DC USA; ^3^ Centre for Outcomes Research and Effectiveness, Research department of Clinical, Educational and Health Psychology University College London London UK; ^4^ Addiction Research Group, Norwich Medical School University of East Anglia Norwich UK; ^5^ Tobacco Research and Treatment Center, Department of Medicine, Massachusetts General Hospital Harvard Medical School Boston MA USA

**Keywords:** E‐cigarettes, flavours, nicotine, smoking cessation, systematic review, tobacco

## Abstract

**Aims:**

To estimate associations between e‐cigarette flavour and smoking cessation and study product use at 6 months or longer.

**Methods:**

Secondary analysis of data from a living systematic review, with meta‐analyses and narrative synthesis, incorporating data up to January 2022. Included studies provided people who smoked combustible cigarettes with nicotine e‐cigarettes for the purpose of smoking cessation compared with no treatment or other stop smoking interventions. Measurements included smoking cessation and study product use at 6 months or longer reported as risk ratios (RR) with 95% confidence intervals (CI); and flavour use at any time‐points.

**Results:**

We included 16 studies (*n* = 10 336); 14 contributed to subgroup analyses and 10 provided participants with a choice of e‐cigarette flavour. We judged nine, five and two studies at high, low and unclear risk of bias, respectively. Subgroup analyses showed no clear associations between flavour and cessation or product use. In all but one analysis, tests for subgroup differences resulted in *I* ^2^ values between 0 and 35%. In the comparison between nicotine e‐cigarettes and nicotine replacement therapy (NRT) (*I* ^2^ = 65.2% for subgroup differences), studies offering tobacco flavour e‐cigarettes showed evidence of a greater proportion of participants still using at 6 months or longer (RR = 3.81; 95% CI = 1.45–10.05; *n* = 1181; *I* ^2^ = 84%), whereas there was little evidence for greater 6‐month use when studies offered a choice of flavours (RR = 1.44; 95% CI = 0.80–2.56; *n* = 454; *I* ^2^ = 82%). However, substantial statistical heterogeneity within subgroups makes interpretation of this result unclear. In the 10 studies where participants had a choice of flavours, and this was tracked over time, some switching between flavours occurred, but there were no clear patterns in flavour preferences.

**Conclusions:**

There does not appear to be a clear association between e‐cigarette flavours and smoking cessation or longer‐term e‐cigarette use, possibly due to a paucity of data. There is evidence that people using e‐cigarettes to quit smoking switch between e‐cigarette flavours.

## INTRODUCTION

E‐cigarettes (EC) are a relatively new and popular approach to quitting smoking. The most recent update of our Cochrane living systematic review of ‘Electronic cigarettes for smoking cessation’ shows moderate‐certainty evidence that more people successfully quit smoking using nicotine EC than using nicotine replacement therapy (NRT) or non‐nicotine EC [1]. EC are available in a variety of flavours that can be matched to either a person’s favoured cigarette type; that is, tobacco or menthol, or something completely different, such as fruits, candies or desserts. There are ongoing policy debates about restricting flavour options, particularly as a mechanism to prevent youth vaping. While population surveys have attempted to examine whether EC flavours affect smoking cessation [2, 3, 4], there is very little evidence available on how EC flavours influence quitting in clinical trials, and this is not currently addressed in our Cochrane review.

There are many reasons to think that flavour might impact the effects of ECs for quitting smoking. Using a flavour that matches a user’s flavour of combustible cigarettes could hypothetically boost the likelihood of successful quitting if the people using them are less likely to miss their combustible cigarettes and thus less likely to relapse. However, there is also the possibility that using an EC flavour different to a person’s usual cigarettes could both increase the novelty and desirability of the product and also reinforce the established addiction less than flavours associated with cigarettes, thereby reducing cigarette dependence. A systematic review, including any type of study that analysed differences between EC flavours published between 2007 and August 2020, found evidence that people who smoked combustible cigarettes and used non‐tobacco‐flavoured e‐liquids were more likely to have reduced or quit smoking than those using tobacco or unflavoured e‐liquids [5]. Simply having a choice of flavour options to switch between could also make EC a more desirable quitting aid, able to meet a person’s changing preferences throughout their quit attempt. The previously mentioned systematic review found that EC users valued the ability to switch between flavours and it was one of the main reasons given for EC use; following health and smoking cessation [5]. A UK cross‐sectional survey found that people typically started out using tobacco‐flavoured e‐cigarettes and transferred to sweet or food‐flavoured products [6].

However, the potential benefits of a range of flavour options for smoking cessation must be balanced against concerns that the availability of flavours, such as fruits, candies and desserts, make EC more desirable to people who have never smoked, especially young people, and that this will result in more people using EC recreationally as opposed to as a quitting aid. A recent review provides some evidence that people under the age of 18 years do enjoy flavoured EC products and have a preference for fruit and other sweet flavours [7]. This has led to bans, or consideration of bans, on the sale of particular EC flavours in some jurisdictions [8]. Another important consideration is that the use of flavoured, as opposed to unflavoured, EC products may increase the length of time that people continue to use EC after making a smoking quit attempt. This could have positive implications if longer‐term EC use reduces the risk of relapse to smoking combustible cigarettes, but could also be a cause for concern if long term use leads to health harms.

As part of the ongoing discussions feeding into policy decisions, here we investigate whether EC flavours are associated with tobacco smoking quit success or longer‐term use of EC in adults when provided as stop smoking aids in intervention studies of EC for smoking cessation that meet the eligibility criteria for our Cochrane review [1]. Our objectives were as follows:

1. To investigate whether the effectiveness of using nicotine EC to stop smoking in comparison to smoking cessation pharmacotherapies, non‐nicotine EC, behavioural support only or no support is associated with flavour of EC used; and
2. To investigate whether the long‐term (6+ months) use of study product is associated with flavour of nicotine EC used.

## METHODS

### Searches, screening and data extraction

This analysis builds on our living systematic review of EC for smoking cessation; therefore, more information on search methods, eligibility criteria and data extraction is available in that review, as well as in the Supporting information [1]. Our methods for these particular analyses were pre‐registered on Open Science Framework (https://osf.io/HPBYW/). Briefly, we include randomized controlled trials (RCTs) or randomized cross‐over trials in which people who smoke combustible cigarettes are randomized to EC or any control condition. In addition, we include uncontrolled intervention studies in which all participants receive an EC intervention, although these studies are not included in our meta‐analyses. All studies must report abstinence from cigarettes at 6 months or longer, data on safety markers at 1 week or longer, or both, to be included. However, specific to the investigation here we only carried out further investigation of studies that were identified in our searches up to January 2022 and provided data on at least one of the following outcomes:

- long‐term cessation of combustible cigarettes (at 6‐month follow‐up or longer; we also refer to this as ‘abstinence’ in the text which pertains specifically to abstinence from combustible cigarettes)
- proportion of people still using study product (EC or comparator intervention) at longest follow‐up (at 6‐month follow‐up or longer).

Although we carry out screening in duplicate for our main review, a single reviewer carried out the second stage of eligibility screening for the analyses reported here. Data extraction was conducted in duplicate as part of the parent review; a single reviewer then went back through the eligible studies and extracted relevant information on flavours, which was checked by a second reviewer. Flavours were categorized into subgroups: tobacco only; menthol/mint only; sweet only (including fruit, candy and dessert flavours); unflavoured only; choice of tobacco or menthol/mint; choice of tobacco, menthol/mint or sweet; and unspecified.

Risk‐of‐bias judgements follow those of the parent review [1]; studies were judged to be at low risk of bias overall if judged at low risk across all domains assessed, at high risk of bias if assessed at high risk in one or more domain, and at unclear risk where no domains were judged to be at high risk but at least one was judged to be at unclear risk.

### Analyses

To investigate associations between flavours and our outcomes of interest, we subgrouped existing meta‐analyses from our Cochrane review by the flavours of EC used in the included studies, for the following primary comparisons:

- nicotine EC versus NRT
- nicotine EC versus varenicline
- nicotine EC versus non‐nicotine EC
- nicotine EC versus behavioural support only or no intervention.

We updated our existing analyses using Cochrane’s Revman version 5.4 software where there were sufficient studies and data, and investigated subgroup differences using *I* ^2^ for subgroup differences (where there were more than one study and subgroup in an analysis). We also examined the pooled estimates for each subgroup and judged whether their interpretation differed across groups. We calculated pooled effect estimates as risk ratios (RR) and 95% confidence intervals (CI). In the parent review [1] analyses are carried out using fixed‐effects methods, and that is what we planned to do in the protocol for this review. For this paper, we present these updated fixed effects analyses in the Supporting information. However, in the main text we present *post‐hoc* random‐effects analyses, as requested by the journal. Had any of our analyses included 10 or more studies we would have investigated potential publication bias using funnel plots, in line with Cochrane guidance [9].

Where studies offered participants a range of flavours, a single reviewer extracted any information reported on participants’ flavour choices. We also planned to extract the results of any analyses authors had carried out of their outcomes by flavours chosen and synthesize these narratively. None of the studies reported these types of analyses; therefore, we contacted the authors of all of the papers that reported a choice of flavours to determine if they were able to provide any further information. Where this information was available, we report results narratively and in descriptive graphs.

## RESULTS

### Included studies

Throughout January 2022, our literature searches identified 67 studies eligible for inclusion in our Cochrane living review of electronic cigarettes for smoking cessation. One of these studies was excluded from the current investigation as the investigators did not provide participants with EC [10]. A further 28 were excluded as they did not report on our outcomes of interest, and four were excluded as they did not provide any information on EC flavour and we were not successful in obtaining further information from the investigators. Of the remaining 34 studies, 18 were neither RCTs (and so ineligible for our subgrouped meta‐analyses) nor provided participants with a choice of EC flavour. This left 16 studies (see Supporting information for flow diagram); 14 of these were RCTs eligible for inclusion in relevant meta‐analyses and 10 were studies that reported providing participants with a choice of EC flavour (some studies were included in both types of synthesis). From the latter we attempted to extract further information on participant choice and the impact of flavour on our outcomes of interest. Nine of the eligible studies were judged to be at high risk of bias overall, two at unclear risk and five at low risk (for further information on risk of bias judgements see the Supporting information). Table 1 includes brief summary information on these studies. We did not identify any completed studies that randomized participants to different flavour choices and reported data on our pre‐specified outcomes.

**TABLE 1:** Characteristics of included studies.

Study ID	Device type	Total *N* baseline	Flavours provided	Comparison (C) or single arm (S)	Study design	Length of FU (months)	Overall risk of bias judgement	Country	Population characteristics
Begh 2021 [11]	Refillable	325	Choice of sweet, tobacco or menthol	C (EC versus standard care)	RCT	8	High	UK	People who smoke combustible cigarettes with no plans to stop
Bullen 2013 [12]	Cig‐a‐like	657	Tobacco only	C (EC versus nicotine patches versus placebo EC)	RCT	6	Low	NZ	People who smoke combustible cigarettes and willing to quit
Caponnetto 2013 [13]	Cig‐a‐like	300	Tobacco only	C (EC versus lower nicotine EC versus non‐nicotine EC)	RCT	12	Unclear	Italy	People who smoke combustible cigarettes
Cobb 2021 [14]	Cartridge	520	Choice of tobacco or menthol	C (EC nicotine 2 strengths; non‐nicotine EC; cigarette substitute)	RCT	6	Low	USA	People who smoke combustible cigarettes
Dawkins 2020 [15]	Refillable	80	Choice of sweet, tobacco or menthol	C (EC versus UC)	Prospective cohort	6	High	UK	People who smoke combustible cigarettes Recruitment at homeless centres
Eisenberg 2020 [16]	Cig‐a‐like	376	Tobacco only	C (EC + counselling versus non‐nicotine EC + counselling versus counselling only)	RCT	6	Low	Canada	People who smoke combustible cigarettes and motivated to quit
Ely 2013 [17]	Cig‐a‐like	48	Choice of sweet, tobacco or menthol	S (all used EC)	Prospective cohort	6	High	USA	People who want to quit combustible cigarettes or switch to EC
Hajek 2019 [18]	Refillable	886	Tobacco only	C (EC versus NRT)	RCT	12	Low	UK	People who smoke combustible cigarettes
Halpern 2018 [19]	Cig‐a‐like	6006	Choice of sweet, tobacco or menthol	C [usual care (UC); UC + EC; UC + EC + NRT + bupropion or varenicline]; UC + EC + NRT + bupropion or varenicline + incentives; as before plus financial incentive)	RCT	12	High	USA	People who smoke and employees and their spouses that used Vitality wellness programmes
Holliday 2019 [20]	Refillable	80	Choice of sweet, tobacco, mint/menthol or unflavoured	C (EC versus no intervention)	RCT	6	High	UK	People who smoke combustible cigarettes with periodontitis
Lee 2018 [21]	Cig‐a‐like	30	Tobacco only	C (EC versus nicotine patches)	RCT	6	Low	USA	People who smoke and presented to the anaesthesia preoperative clinic for elective surgery 3 or more days before surgery
Lucchiari 2020 [22]	Cig‐a‐like	210	Tobacco only	C (nicotine EC versus non‐nicotine EC)	RCT	12 but data only available at 6	High	Italy	Participants are aged 55 years or more and have smoked at least 10 combustible cigarettes a day for the past 10 years
Myers Smith 2021 [23]	Refillable	135	Choice of sweet, tobacco or menthol	C (EC versus NRT)	RCT	6	Low	UK	People who smoke combustible cigarettes and find quitting difficult
Polosa 2015 [24]	Refillable	71	Choice of sweet, tobacco or menthol	S (all used EC)	Prospective cohort	12	High	Italy	People who smoke combustible cigarettes, making first purchase at vape shop
Pulvers 2020 [25]	Pod	186	Choice of sweet, tobacco or menthol	C (EC versus no intervention)	RCT	6	High	USA	African American and Latinx people who smoke combustible cigarettes
Russell 2021 [26]	Pod	426	Choice of sweet, tobacco or menthol	C (NRT; EC with nicotine salt e‐liquid pods; EC with freebase nicotine e‐liquid pods)	RCT	6	Unclear	UK	People who smoke combustible cigarettes

EC = electronic cigarette; NRT = nicotine replacement therapy.

### Associations between flavours and outcomes of interest

**FIGURE 1:**
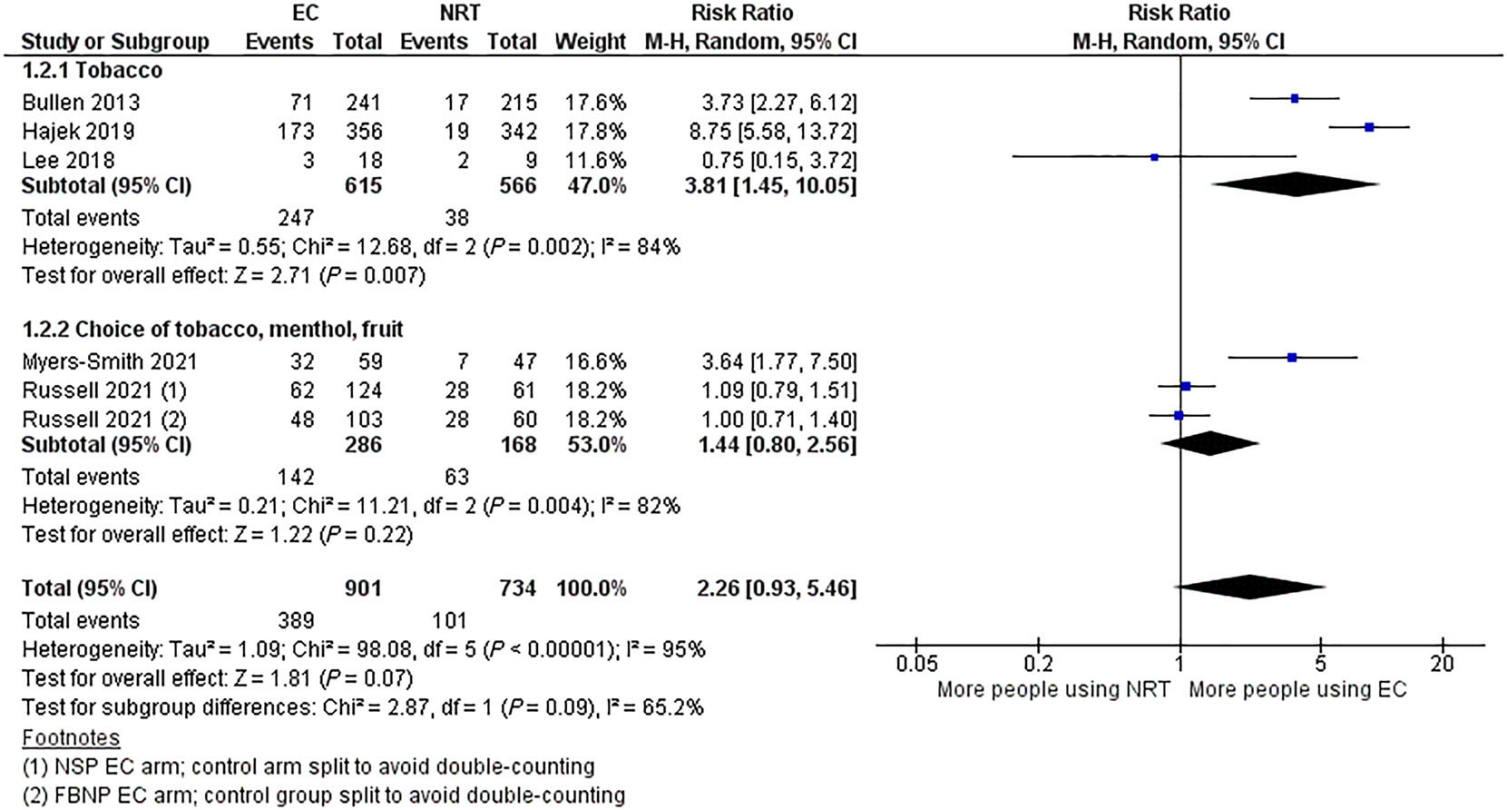
Study product use at 6 months or longer, electronic cigarette (EC) versus nicotine replacement therapy (NRT). FBNP = free base nicotine pods; NSP = nicotine salt pods

For the majority of the subgrouped meta‐analyses that we were able to conduct, subgrouping by our specified flavour categories showed no clear evidence of effect moderation (see Table 2 for summaries of feasible subgroup analyses). The only analysis where there was an *I* ^2^ suggesting substantial statistical heterogeneity between groups (*I* ^2^ = 65.2%) was for the comparison ‘nicotine EC versus NRT’ and the outcome of long‐term product use (Figure 1). Three studies were included in a ‘tobacco’‐flavour subgroup [12, 18, 21] and two studies in a ‘choice of tobacco, menthol/mint or sweet’ group [23, 26]. The ‘tobacco’ subgroup provided evidence that more participants were using EC at long‐term follow‐up than were using NRT; whereas the ‘choice’ subgroup had a substantially smaller point estimate, favouring higher long‐term EC use with CIs, also encompassed the potential for higher long‐term NRT use as well as no difference in the product use between study groups. However, this finding should be treated with caution, as there was substantial statistical heterogeneity within subgroups (*I* ^2^ = 84% for ‘tobacco’ subgroup; *I* ^2^ = 82% for ‘choice of tobacco, menthol/mint, sweet’ subgroup) and other differences between the studies could have been driving the apparent differences in effects. It is also important to note that all of our analyses were limited by imprecision due to small numbers of events within analyses and subgroups, and future eligible studies could change the interpretation of subgroup differences (see Supporting information for additional forest plots, as well as the results of the subgrouping of fixed‐effects analyses). Due to the small numbers of studies (< 10) in each of our analyses it was not appropriate to investigate publication bias using funnel plots.

**TABLE 2:** Results from meta‐analyses subgrouped by flavour (random‐effects).

Comparison	Outcome	Number of studies	*I* ^2^ for subgroup differences	*P*‐value for subgroup differences
Nicotine EC versus NRT	Smoking cessation	3 tobacco only; 2 choice of tobacco, menthol or sweet	0	0.91
	Study product use (Figure 1)		65.2	0.09
Nicotine EC versus non‐nicotine EC	Smoking cessation	4 tobacco only; 1 choice of tobacco or menthol	35.0	0.21
	Study product use	2 tobacco only; 1 choice of tobacco or menthol	0	0.86
Nicotine EC versus behavioural support only or no support	Smoking cessation	2 tobacco only; 4 choice of tobacco, menthol or fruit	0	0.58

EC = electronic cigarette; NRT = nicotine replacement therapy.

### Studies offering a choice of flavours

**FIGURE 2:**
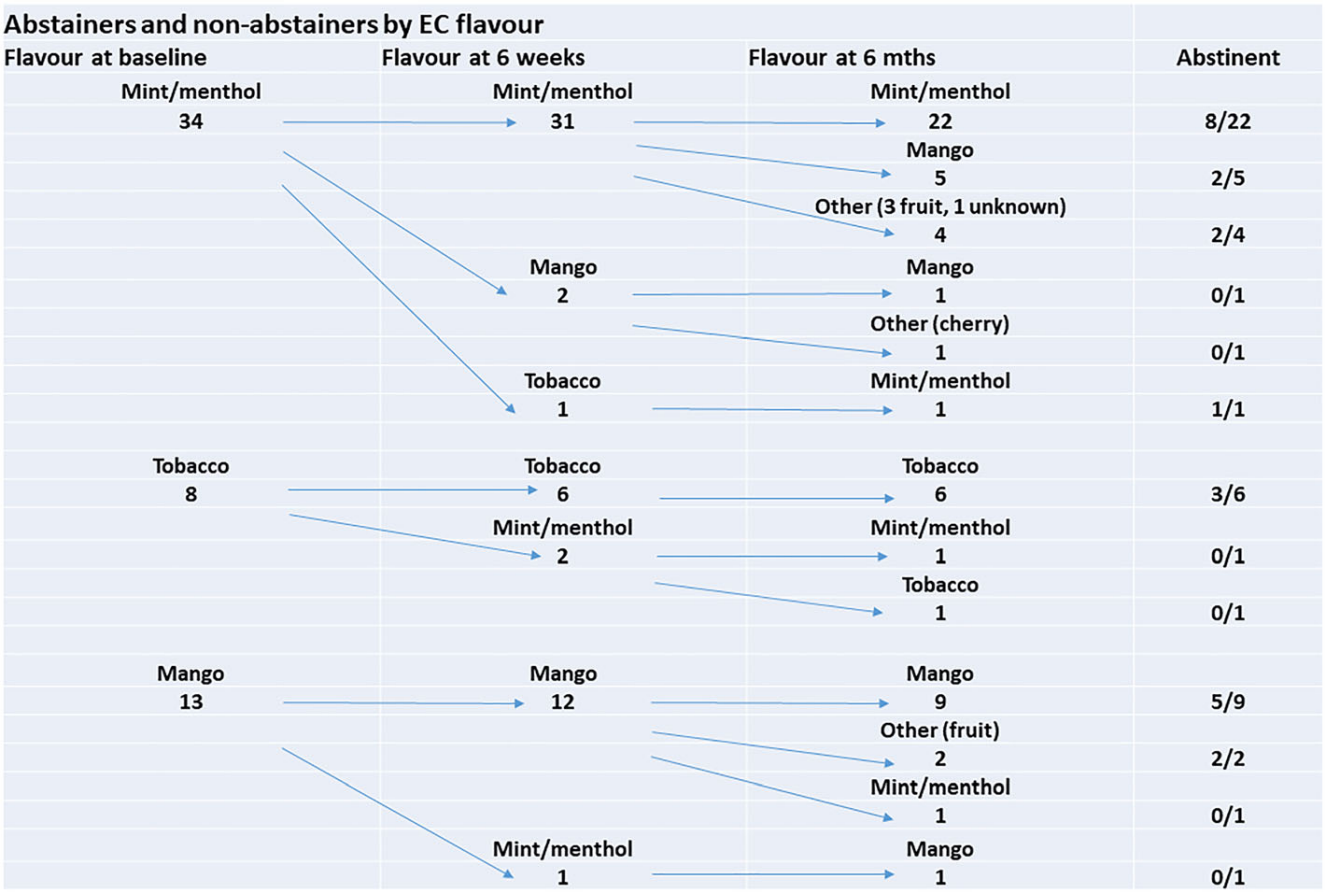
Electronic cigarette (EC) flavour use over time among participants in Pulvers 2020 [25]. Arrows illustrate the flow of flavour choice and switching behaviour (only including participants that provided data at 6‐month follow‐up). At baseline and 6‐week follow‐up, participants were provided with mango, mint, menthol or tobacco flavours. At 6‐month follow‐up, participants were self‐sourcing flavours, so additional flavours were being used, as specified

Table 3 reports the information we extracted on the flavours offered by the 10 studies that provided participants with a choice of flavours. All the studies provided participants with a choice of menthol/mint, tobacco or a sweet flavour (usually fruit), apart from one, which only gave the option of tobacco and menthol [14]. Participant preferences differed among studies, with some seeing a higher popularity of fruit (or other sweet/dessert) flavours over tobacco and menthol flavours [11, 15, 23], one earlier study finding a clear preference for tobacco flavour [24], another for menthol/mint [25] and others seeing a less clear demarcation in preferences. Using individual participant data supplied by one of the author teams, we were also able to map the flavour‐switching behaviour of participants in the study (see Figure 2) [25]. A substantial minority of participants switched the flavour of EC they used during the course of the study. The numbers using menthol flavour decreased slightly over time, the numbers using mango or other fruit flavours increased and those using tobacco remained stable; absolute numbers were small for all samples. Another included RCT found that the proportion of participants using fruit flavours had reduced at 8 months and the proportions using menthol and tobacco had increased [11].

**TABLE 3:** Flavours available and participant choices in studies that offered a range of flavours.

Study ID	Country	Flavour choices available	Choice data
Begh 2021 [11]	UK	Initially provided a starter kit including (blueberry, mixed fruit and menthol). Participants could then purchase flavours of their choice	At 2 months (*N* = 111): blueberry *n* = 15 (13.5%); forest fruit *n* = 28 (25.25%); strawberry *n* = 5 (4.5%), other fruit flavours *n* = 20 (18%); menthol *n* = 10 (9%), tobacco *n* = 21 (18.9%), unflavoured *n* = 1 (0.9%), other flavours (blackjack, bubblegum, CBD oil, coffee, ginger, liquorice, mint, nicotine, toffee, vanilla) *n* = 11 (9.9%) At 8 months (*N* = 32): blueberry *n* = 6, (18.8%); forest fruit *n* = 4 (12.5%), other fruit flavour *n* = 1 (3.1%), menthol *n* = 11 (34.4%), tobacco *n* = 10 (31.3%)
Cobb 2021 [14]	USA	Tobacco or menthol (participants selected their preference at randomization)	Not reported
Dawkins 2020 [15]	UK	Tobacco, fruit or menthol (participants were ‘permitted to switch between flavours’)	Across the duration of the study, 318 bottles of fruit flavoured 10 ml e‐liquid were dispensed; 155 bottles of menthol and 133 bottles of tobacco.
Ely 2013 [17]	USA	Tobacco, menthol, various fruit, various dessert	Not reported
Halpern 2018 [19]	USA	Tobacco, menthol, various fruit	Not reported
Holliday 2019 [20]	UK	Initially provided 2‐week supply of e‐liquid, with a choice of tobacco, mint, cherry flavours or unflavoured. Participants could then purchase flavours of their choice	Total *N* = 39. Cherry only *n* = 4 (10%); mint only *n* = 8 (21%); mint and cherry *n* = 6 (15%); tobacco only *n* = 5 (13%); tobacco and cherry *n* = 2 (5%); tobacco and mint *n* = 9 (23%); unflavoured only *n* = 0; unflavoured and tobacco *n* = 3 (8%); unflavoured and mint *n* = 1 (3%); unflavoured and cherry *n* = 1 (3%)
Myers‐Smith 2021 [23]	UK	Participants independently obtained e‐liquids of their choice and were encouraged to try different flavours	At 1 week (*N* = 49): fruit: *n* = 21; sweet: 5; energy/soft drink: 2; coffee: 3; menthol/mint: 8; tobacco: 13; unknown other: 6 (multiple flavours used by some) At 6 months (*N* = 31): fruit: 18; sweet: 2; energy/soft drink: 2; menthol/mint: 5; tobacco: 6; raspberry and mint: 1; coffee and coconut: 1
Polosa 2015 [24]	Italy	‘Large selection of flavours’	Baseline (*N* = 71): fruit *n* = 4 (5.6%); mint *n* = 7 (9.9%); tobacco *n* = 57 (80.3%); unknown other *n* = 3 (4.2%) At 12 months (*N* = 49): fruit *n* = 2 (4.1%); mint *n* = 5 (10.2%); tobacco *n* = 36 (73.5%); other flavours (cola, coffee, dessert/cakes/cookies, cocktail, mixed berry, mint) *n* = 6 (12.3%).
Pulvers 2020 [25]	USA	Menthol, mint, mango, tobacco	Baseline (*N* = 125): mango *n* = 35 (28%); menthol *n* = 44 (35.2%); mint *n* = 24 (19.2%); tobacco *n* = 22 (17.6%) At 2 and 6 weeks (*N* = 113): mango *n* = 36 (31.9%); menthol *n* = 39 (34.5%); mint *n* = 17 (15%); tobacco *n* = 21 (18.6%).
Russell 2021 [26]	UK	Participants independently obtained e‐liquids of their choice and were encouraged to try different flavours	Not reported

**FIGURE 3:**
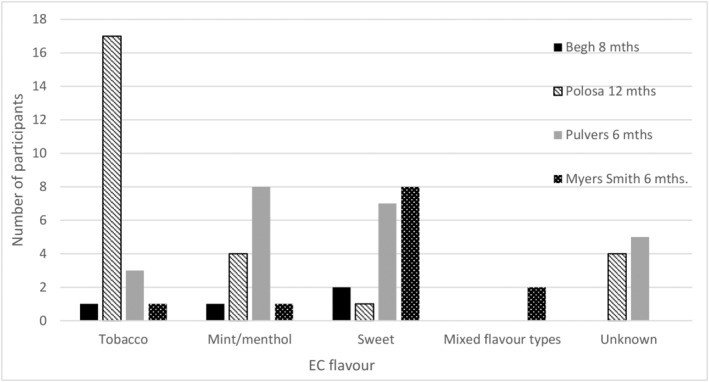
Electronic cigarette (EC) flavour use among people abstinent from combustible cigarettes at longest follow‐up. In the mixed flavour types category, one participant was using both coconut and coffee flavoured e‐liquids and one participant was using both raspberry and mint flavours

**FIGURE 4:**
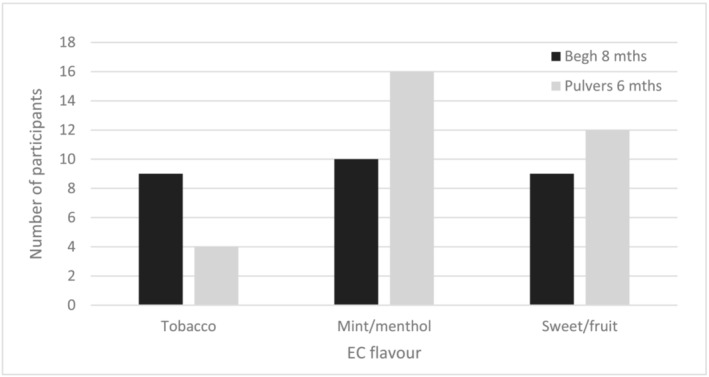
Electronic cigarette (EC) flavour use among people continuing to use combustible cigarettes at longest follow‐up

As none of these 10 studies reported any analyses of smoking cessation or long‐term product use moderated by flavour of EC, we contacted the author teams to see if they could provide any further information. Four of them provided us with some additional information related to flavour use and smoking abstinence [11, 23, 24, 25]. The flavour most commonly used by abstainers varied between studies (see Figure 3). In two UK studies the majority of people who were abstinent were using sweet flavours, with very similar numbers using menthol/mint and tobacco [11, 23]. In a US study, the participants who chose sweet (mango) flavour at baseline were most likely to be abstinent at 6‐month follow‐up (54% quit versus 38% menthol/mint and 37% tobacco; see Figure 2), although most abstainers were using sweet/fruit, followed closely by menthol/mint flavours at follow‐up [25]. In an Italian study, sweet flavours appeared to be used the least by abstainers at follow‐up, with tobacco the most popular flavour [24]. Two of the studies also provided the number of people using each EC flavour who were not abstinent at 8‐ and 6‐month follow‐ups, respectively (Figure 4) [11, 25]. Among the non‐abstinent group flavour use was evenly matched in one study, with nine participants using tobacco, 10 using mint and nine using sweet flavours [11]. However, in the other group, tobacco flavour seemed to be less popular at follow‐up than menthol and fruit flavours (similar to their abstinent participants), with mint/menthol the most popular [25]. As with our previous analyses, it is important to treat the data on abstinence and flavours with caution, as all of the studies that supplied data were relatively small.

## DISCUSSION

This paper reports findings from syntheses conducted as an extension to our Cochrane living systematic review of ‘Electronic cigarettes for smoking cessation’ [1]. Our aim was to investigate any moderating effect of EC flavour on the success of EC as a smoking cessation aid and the likelihood of EC being used long‐term. Subgrouping the analyses from our original review by flavour of EC offered did not provide any clear evidence that cessation or long‐term product use was associated with the flavours provided. However, these findings are based on small numbers of studies and participants and are subject to confounding, thus are likely to change as more evidence becomes available.

Participants’ flavour preferences differed across studies, with some studies showing similar levels of use across flavours, some showing a preference for sweet/fruit flavours, another for tobacco flavour and another for menthol/mint. In all the studies that looked at flavour use over time, there seemed to be some flavour‐switching; however, in most cases it was difficult to distinguish the extent of this due to a lack of individual participant data. In the one US study where we could track individual use, there was notable experimentation with different flavours in some participants, although others used the same flavour throughout [25].

A subset of studies provided some data on the flavours used by participants who were ultimately abstinent from tobacco at long‐term follow‐up; no consistent flavour pattern was observed across studies [11, 23, 24, 25]. However, these findings should again be treated with caution due to the small number of participants, the fact that flavour use was provided at one time‐point only, and switching was likely to have occurred in some people.

Our approach is based on data from a high‐quality, established systematic review [1]. The searches and processes carried out to identify studies are thorough and involve searching for unpublished, as well as published literature, in an attempt to minimize bias. Therefore, we have maximized our chances of identifying all of the relevant literature. For pragmatic reasons the screening and data extraction for this substudy wase carried out by a single author, potentially increasing the opportunity for human error. However, the manuscript has been reviewed by all the authors, the majority of whom are experts in the field and authors on the original Cochrane living review, and thus know the included studies well.

As mentioned above, all of the syntheses included in this paper are based on a small number of studies and participants. The number of intervention studies that have provided information on EC flavour use and preference is small, and none of the studies providing a choice of flavours have carried out their own analyses based on these preferences. Therefore, our investigation and conclusions are severely limited by the lack of available primary data, and particularly by a lack of individual participant data. In addition, the one subgrouped meta‐analysis that appeared to show a potential association between long‐term EC use and flavour was also subject to considerable statistical heterogeneity within subgroups, making it difficult to draw meaningful conclusions. At the time of writing, there are no published RCTs that directly compare different EC flavours and observe our outcomes of interest. Consequently, we were only able to explore associations rather than casual relationships.

Other recent literature on EC flavours provides disparate findings, very similar to the studies synthesized here. Data from a large longitudinal cohort study (Population Assessment of Tobacco and Health: PATH) collected in the United States between 2014 and 2016, found that the most popular flavours of EC were fruit, and that younger people more commonly used products of multiple flavours and changed the flavours they used over time [27]; whereas Nielsen scanner data collected on US purchase transactions between 2013 and 2017 found that adult cigarette smokers tended to purchase tobacco flavour EC or e‐liquids the most [28]. In addition, EC consumers appeared to be loyal to their preferred flavour. A New Zealand study, of 32 participants who completed at least four interviews, provided participants with an EC starter kit but required them to source e‐liquids of their choosing [29]. The majority initially selected a tobacco‐flavoured e‐liquid, with the remainder choosing fruit, menthol/mint, dessert/sweet and non‐alcoholic beverage flavours in approximately equal proportions. Most participants were using the same flavour at study exit; however, many also described experimentation towards the beginning of the study. Experimentation was less common in those who chose a tobacco‐flavoured e‐liquid at baseline. Finally, the aforementioned systematic review of any study that investigated differences in EC flavours published up to August 2020 found evidence that flavour preferences had changed over time [5], with a preference for the more traditional cigarette flavours of tobacco and menthol shifting towards sweet flavours. This appeared to be true even in people using combustible cigarettes together with EC and older EC users, although tobacco was used more in these groups than in younger users, people who used to smoke or people with no history of combustible cigarette use. The authors hypothesize that the shift in the popularity of sweet flavours could be the result of a change in preference or could reflect the increased availability of novel flavours on the market.

While conducting our living Cochrane review we have identified 59 ongoing intervention studies of EC for smoking cessation, which are potentially relevant for inclusion when complete; three of these are RCTs that plan to directly compare different EC flavours [30, 31, 32]. NCT04708106 plans to compare tobacco flavour EC, menthol flavour EC and no intervention [30]. NCT04090879 plans to compare tobacco flavour EC with a choice of EC flavours [31], and NCT05023096 plans to compare menthol and tobacco EC, tobacco EC only and unflavoured EC [32]. In two further ongoing studies, investigators will test whether allowing participants to personalize the flavour of their EC e‐liquid has any moderating effects on tobacco cigarette smoking [33, 34]. These studies are all poised to offer further, valuable information, although flavour choice among them remains limited primarily to tobacco and menthol options. There is currently little evidence available on the potential toxicity of different e‐cigarette flavours, which we recognize is difficult to assess due to the large number of flavourings in use, and the multiple mediating and confounding factors, such as device type [35]. As more head‐to‐head trials comparing flavours become available we expect the monitoring of relative differences in safety outcomes to be more likely. Up‐to‐date individualized information on toxicity should be taken into account when considering the availability and use of particular flavours.

In addition, due to the limited and aggregated data included in this review, we were unable to examine variables that may be mediating or confounding the relationship between flavour use and smoking cessation in more depth; for example, nicotine concentration, device power, amount of e‐liquid used, number of puffs taken. The investigation of these variables in future studies could help us to understand the role flavours play in quitting smoking and find reasons for the heterogeneity between study effects identified in this review.

In conclusion, at the time of writing, intervention studies investigating EC for smoking cessation for 6 months or longer provide very little information on the popularity of EC flavours used and any potential impact of these on smoking cessation and long‐term product use. Relevant studies are ongoing, but the range of flavour choices offered are limited. Due to current uncertainties regarding the relative benefits of flavours, future studies should aim to explore a broad range of flavours in order to inform ongoing policy debate and decisions with regard to the availability of EC flavours. Studies should also report data on important outcomes broken down by flavour type and exploring potential mediating and confounding factors, such as nicotine concentration, device type, e‐liquid use and number of puffs. Long‐term RCTs directly comparing the effects of different flavours on smoking and vaping behaviours, as well as exploring product safety, are particularly needed. Based on the evidence that flavour experimentation takes place during studies, collecting detailed information concerning the flavours used by individual participants throughout the duration of studies would be beneficial. It is possible that particular flavours are favoured for achieving abstinence and others for maintaining it. Finally, although the varied flavour preferences among studies preclude clear conclusions on user preferences, this may reflect genuine individual differences among EC users. If so, this has implications for the popularity of EC as a smoking aid if the range of flavours offered were to be limited as a result of government policies.

## DECLARATION OF INTERESTS

N.A.R. has received royalties from UpToDate, Inc. for chapters on electronic cigarettes. Outside the topic of e‐cigarettes, she has consulted for and received a research grant from Achieve Life Sciences.

## AUTHOR CONTRIBUTIONS

**Nicola Lindson:** Conceptualization; data curation; formal analysis; funding acquisition; investigation; methodology; project administration; resources; software; supervision; validation; visualization; writing‐original draft; writing‐review and editing. **Ailsa R Butler:** Conceptualization; data curation; formal analysis; funding acquisition; investigation; methodology; project administration; resources; software; supervision; validation; visualization; writing‐original draft; writing‐review and editing. **Alex Liber:** Conceptualization; data curation; formal analysis; funding acquisition; investigation; methodology; project administration; resources; software; supervision; validation; visualization; writing‐original draft; writing‐review and editing. **David Levy:** Conceptualization; data curation; formal analysis; funding acquisition; investigation; methodology; project administration; resources; software; supervision; validation; visualization; writing‐original draft; writing‐review and editing. **Phoebe Barnett:** Data curation; formal analysis; investigation; methodology; project administration; resources; software; supervision; validation; visualization; writing‐original draft; writing‐review and editing. **Annika Theodoulou:** Conceptualization; data curation; formal analysis; funding acquisition; investigation; methodology; project administration; resources; software; supervision; validation; visualization; writing‐original draft; writing‐review and editing. **Caitlin Jade Notley:** Conceptualization; data curation; formal analysis; funding acquisition; investigation; methodology; project administration; resources; software; supervision; validation; visualization; writing‐original draft; writing‐review and editing. **Nancy Rigotti:** Conceptualization; data curation; formal analysis; funding acquisition; investigation; methodology; project administration; resources; software; supervision; validation; visualization; writing‐original draft; writing‐review and editing. **Jamie Hartmann‐Boyce:** Conceptualization; data curation; formal analysis; funding acquisition; investigation; methodology; project administration; resources; software; supervision; validation; visualization; writing‐original draft; writing‐review and editing.

## Supporting information


**Data S1.** Supporting Information

## References

[1] Hartmann‐Boyce J , McRobbie H , Butler AR , Lindson N , Bullen C , Begh R , et al. Cochrane Database Syst Rev. 2021;(8):CD010216. 10.1002/14651858.CD010216.pub4 PMC8094228 33052602

[2] Glasser AM , Vojjala M , Cantrell J , Levy DT , Giovenco DP , Abrams D , et al. Patterns of e‐cigarette use and subsequent cigarette smoking cessation over 2 years (2013/2014–2015/2016) in the population assessment of tobacco and health study. Nicotine Tob Res. 2021;23:669–77. 32939555 10.1093/ntr/ntaa182 PMC7976933

[3] Harlow AF , Fetterman JL , Ross CS , Robertson RM , Bhatnagar A , Benjamin EJ , et al. Association of device type, flavours and vaping behaviour with tobacco product transitions among adult electronic cigarette users in the USA. Tob Control. 2022;31:e10–7. 33479031 10.1136/tobaccocontrol-2020-055999 PMC8292448

[4] Friedman AS , Xu S . Associations of flavored e‐cigarette uptake with subsequent smoking initiation and cessation. JAMA Netw Open. 2020;3:6. 10.1001/jamanetworkopen.2020.3826 PMC7275248 32501490

[5] Gades MS , Alcheva A , Riegelman AL , Hatsukami DK . The role of nicotine and flavor in the abuse potential and appeal of electronic cigarettes for adult current and former cigarette and electronic cigarette users: a systematic review. Nicotine Tob Res. 2022;24:1332–43. 35305014 10.1093/ntr/ntac073 PMC9356694

[6] Gentry SV , Ward E , Dawkins L , Holland R , Notley C . Reported patterns of vaping to support long‐term abstinence from smoking: a cross‐sectional survey of a convenience sample of vapers. Harm Reduct J. 2020;17:1–9. 33023583 10.1186/s12954-020-00418-8 PMC7541214

[7] Notley C , Gentry S , Cox S , Dockrell M , Havill M , Attwood AS , et al. Youth use of e‐liquid flavours—a systematic review exploring patterns of use of e liquid flavours and associations with continued vaping, tobacco smoking uptake, or cessation. Addiction. 2022;117:1258–72. 34784651 10.1111/add.15723 PMC9299186

[8] Klein DE , Chaiton M , Kundu A , Schwartz R . A literature review on international e‐cigarette regulatory policies. Curr Addict Rep. 2020;7:509–19.

[9] Higgins JP , Thomas J , Chandler J , Cumpston M , Li T , Page MJ , et al. Cochrane Handbook for Systematic Reviews of Interventions Hoboken, NJ: John Wiley & Sons; 2019. 10.1002/14651858.ED000142 PMC10284251 31643080

[10] Martinez U , Simmons VN , Sutton SK , Drobes DJ , Meltzer LR , Brandon KO , et al. Targeted smoking cessation for dual users of combustible and electronic cigarettes: a randomised controlled trial. Lancet Public Health. 2021;6:e500–9. 34175001 10.1016/S2468-2667(20)30307-8 PMC8281505

[11] Begh R , Coleman T , Yardley L , Barnes R , Naughton F , Gilbert H , et al. Examining the effectiveness of general practitioner and nurse promotion of electronic cigarettes versus standard care for smoking reduction and abstinence in hardcore smokers with smoking‐related chronic disease: protocol for a randomised controlled trial. Trials. 2019;20:1–16. 31779689 10.1186/s13063-019-3850-1 PMC6883522

[12] Bullen C , Howe C , Laugesen M , McRobbie H , Parag V , Williman J , et al. Electronic cigarettes for smoking cessation: a randomised controlled trial. Lancet. 2013;382:1629–37. 24029165 10.1016/S0140-6736(13)61842-5

[13] Caponnetto P , Campagna D , Cibella F , Morjaria JB , Caruso M , Russo C , et al. EffiCiency and safety of an eLectronic cigAreTte (ECLAT) as tobacco cigarettes substitute: a prospective 12‐month randomized control design study. PLOS ONE. 2013;8:e66317. 23826093 10.1371/journal.pone.0066317 PMC3691171

[14] Cobb CO , Foulds J , Yen M‐S , Veldheer S , Lopez AA , Yingst JM , et al. Effect of an electronic nicotine delivery system with 0, 8, or 36 mg/mL liquid nicotine versus a cigarette substitute on tobacco‐related toxicant exposure: a four‐arm, parallel‐group, randomised, controlled trial. Lancet Respir Med. 2021;9:840–50. 33857436 10.1016/S2213-2600(21)00022-9 PMC8349799

[15] Dawkins L , Bauld L , Ford A , Robson D , Hajek P , Parrott S , et al. A cluster feasibility trial to explore the uptake and use of e‐cigarettes versus usual care offered to smokers attending homeless centres in Great Britain. PLOS ONE. 2020;15:e0240968. 33095798 10.1371/journal.pone.0240968 PMC7584191

[16] Eisenberg MJ , Hébert‐Losier A , Windle SB , Greenspoon T , Brandys T , Fülöp T , et al. Effect of e‐cigarettes plus counseling vs counseling alone on smoking cessation: a randomized clinical trial. JAMA. 2020;324:1844–54. 33170240 10.1001/jama.2020.18889 PMC7656286

[17] Ely J . Evaluation of the use of electric cigarettes in a rural smoking cessation program. University of Northern Colorado. 2013. Accessed 10 May 2022. Available at: https://digscholarship.unco.edu/cgi/viewcontent.cgi?article=1001&context=capstones

[18] Hajek P , Phillips‐Waller A , Przulj D , Pesola F , Myers Smith K , Bisal N . et al. A randomized trial of e‐cigarettes versus nicotine‐replacement therapy. N Engl J Med. 2019;380:629–37. 30699054 10.1056/NEJMoa1808779

[19] Halpern SD , Harhay MO , Saulsgiver K , Brophy C , Troxel AB , Volpp KG . A pragmatic trial of e‐cigarettes, incentives, and drugs for smoking cessation. N Engl J Med. 2018;378:2302–10. 29791259 10.1056/NEJMsa1715757

[20] Holliday R , Preshaw PM , Ryan V , Sniehotta FF , McDonald S , Bauld L , et al. A feasibility study with embedded pilot randomised controlled trial and process evaluation of electronic cigarettes for smoking cessation in patients with periodontitis. Pilot Feasibil Stud. 2019;5:1–14. 10.1186/s40814-019-0451-4 PMC6547559 31171977

[21] Lee SM , Tenney R , Wallace AW , Arjomandi M . E‐cigarettes versus nicotine patches for perioperative smoking cessation: a pilot randomized trial. Peer J. 2018;6:e5609. 30280019 10.7717/peerj.5609 PMC6166615

[22] Lucchiari C , Masiero M , Mazzocco K , Veronesi G , Maisonneuve P , Jemos C , et al. Bnefits of e‐cigarettes in smoking reduction and in pulmonary health among chronic smokers undergoing a lung cancer screening program at 6 months. Addict Behav. 2020;103:106222. 31838445 10.1016/j.addbeh.2019.106222

[23] Myers Smith K , Phillips‐Waller A , Pesola F , McRobbie H , Przulj D , Orzol M , et al. E‐cigarettes versus nicotine replacement treatment as harm reduction interventions for smokers who find quitting difficult: randomized controlled trial. Addiction. 2022;117:224–33. 34187081 10.1111/add.15628

[24] Polosa R , Caponnetto P , Cibella F , Le‐Houezec J . Quit and smoking reduction rates in vape shop consumers: a prospective 12‐month survey. Int J Environ Res Public Health. 2015;12:3428–38. 25811767 10.3390/ijerph120403428 PMC4410194

[25] Pulvers K , Nollen NL , Rice M , Schmid CH , Qu K , Benowitz NL , et al. Effect of pod e‐cigarettes vs cigarettes on carcinogen exposure among African American and Latinx smokers: a randomized clinical trial. JAMA Netw Open. 2020;3:e2026324. 33206193 10.1001/jamanetworkopen.2020.26324 PMC7675102

[26] Russell C , McKeganey , Katsampouris E , Satchwell A , Haseen F . 2021. A randomised community‐based trial of a closed‐system pod e‐vapour product and nicotine replacement therapy for cigarette abstinence and reduction [PH‐353]. Society for Research on Nicotine and Tobacco (SRNT) 2021 Annual Meeting; 24–27 February 2021; Virtual 2021; 230.

[27] Bansal‐Travers M , Rivard C , Silveira ML , Kimmel H , Poonai K , Bernat JK , et al. Factors associated with changes in flavored tobacco products used: Findings from wave 2 and wave 3 (2014–2016) of the Population Assessment of Tobacco and Health (PATH) study. Addict Behav. 2022;130:107290. 35220150 10.1016/j.addbeh.2022.107290 PMC9316535

[28] Zare S , Zheng Y . Consumer preferences for e‐cigarette flavor, nicotine strength, and type: evidence from Nielsen scanner data. Nicotine Tob Res. 2021;23:823–8. 33245356 10.1093/ntr/ntaa238

[29] Blank M‐L , Hoek J . Choice and variety‐seeking of e‐liquids and flavor categories by New Zealand smokers using an electronic cigarette: a longitudinal study. Nicotine Tob Res. 2021;23:798–806. 33748862 10.1093/ntr/ntaa248 PMC8628871

[30] NCT04708106 . Characterization of Product Use in Smokers Switching From Cigarettes to a RELX Electronic Nicotine Delivery System. 2021. Accessed 21 March 2022. Available at: https://clinicaltrialsgov/show/NCT04708106

[31] NCT04090879 . Low Nicotine Content Cigarettes in Vulnerable Populations: Affective Disorders. 2019. Accessed 21 March 2022. Available at: https://clinicaltrialsgov/show/NCT04090879

[32] NCT05023096 . Low Nicotine Content Cigarettes in Vulnerable Populations: Affective Disorders. 2019. Accessed 21 March 2022. Available at: https://clinicaltrialsgov/show/NCT04090879

[33] NCT04092387 . Low Nicotine Content Cigarettes in Vulnerable Populations: Women of Reproductive Age. 2019. Accessed 21 March 2022. Available at: https://clinicaltrialsgov/show/NCT04092387

[34] NCT04092101 . Low Nicotine Content Cigarettes in Vulnerable Populations: Opioid Use Disorder. 2019. Accessed 21 March 2022. Available at: https://clinicaltrialsgov/show/NCT04092101

[35] Barhdadi S , Rogiers V , Deconinck E , Vanhaecke T . Toxicity assessment of flavour chemicals used in e‐cigarettes: current state and future challenges. Arch Toxicol. 2021;95:2879–81. 34021776 10.1007/s00204-021-03080-6

