# Supplementary material for: An exploration of flavours in studies of e‐cigarettes for smoking cessation: secondary analyses of a systematic review with meta‐analyses
Source: Addiction. 2022 Dec 5;118(4):634–45. doi: 10.1111/add.16091 (PMC10952306; doi:10.1111/add.16091)
Supplement: Supplementary file 1 — Data S1. Supporting Information [file ADD-118-634-s001.docx]

# Supplementary material


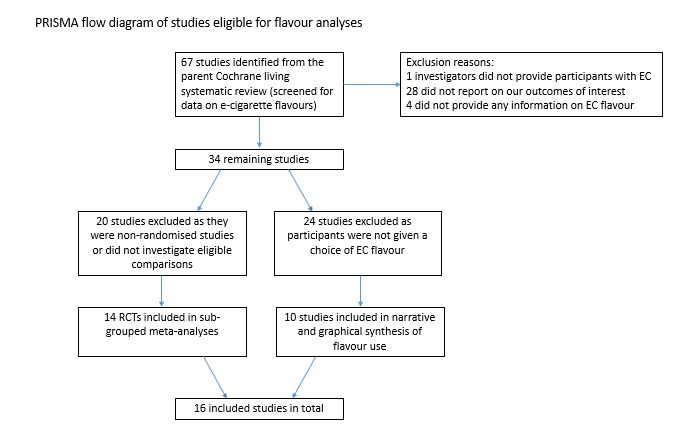


Further detail on relevant Cochrane living review methods^^[[1]](#footnote-1)^^

Study eligibility criteria

In order to be included studies must be randomised controlled trials (including randomised cross-over trials) or uncontrolled intervention studies where all participants are provided with an e-cigarette (either nicotine or non-nicotine containing). Participants must be smoking combustible cigarettes at the study start and e-cigarettes should be provided as an intervention to quit smoking. Where studies have comparison groups these can be other forms of eligible interventions, alternative smoking cessation aids, no intervention or behavioural support. Where behavioural support is provided this should be matched between intervention arms. In order to be included studies should measure at least one of the following:

- Smoking cessation at 6-months follow-up or longer
- Safety (adverse events or serious adverse events) or biomarkers (carbon monoxide, blood pressure, heart rate, blood oxygen saturation, lung function measures, known toxins or carcinogens) at one-week follow-up or longer

Searching, screening and data extraction

We search the following databases monthly:

- Cochrane Tobacco Addiction Group Specialized Register
- Cochrane Central Register of Controlled Trials (CENTRAL)
- MEDLINE (OVID SP)
- Embase (OVID SP)
- PsycINFO (OVID SP)
- [ClinicalTrials.gov](http://clinicaltrials.gov/)
- WHO International Clinical Trials Registry Platform (ICTRP: [www.who.int/ictrp/en/](https://www.who.int/ictrp/en/))

This paper incorporates the results of searches with search date parameters between 2004 (just before e-cigarettes became available) and 1^st^ January 2022. We use the following e-cigarette-related search terms: e‐cig$, electr$ cigar$, electronic nicotine, vape, vapes, vaporizer, vapourizer, vaporiser, vapouriser, vaper, vapers, vaping, electronic nicotine delivery systems, as well as smoking-related and study-related terms.

We carry out eligibility screening in two stages: 1) title and abstract; 2) full-text. At each stage two authors independently screen each citation and where there is disagreement this is resolved through discussion with a third author. Extraction of outcome and risk of bias data is also carried out in duplicate with disagreements resolved through discussion with a third author. Where key information is not available we contact the authors of relevant studies to obtain further information.

Risk of bias assessments

We carry out risk of bias assessments in duplicate using the Cochrane risk of bias 1 tool. Using this approach we assess selection bias, performance bias, detection bias, attrition bias, and reporting bias using six domains. Each domain is given a rating of ‘low’, ‘unclear’ or ‘high’ risk of bias. We judge studies at ‘high’ risk overall if they get at least one judgment of high risk, at ‘low’ risk overall if every individual domain is deemed to be at low risk, and all other studies are judged to be at ‘unclear’ risk overall.

Analysis methods

For dichotomous outcomes, such as smoking cessation and long-term e-cigarette use, we analyse individual study effects and pooled effects by calculating risk ratios (RR) and 95% confidence intervals (CI), using data from the longest follow-up reported. For our smoking cessation outcome, where participants are lost to follow-up we assume they are smoking as is standard in the field. When pooling data across studies is deemed appropriate we use a Mantel‐Haenszel fixed-effect model (decided a priori, in-line with the editorial guidance of the Cochrane Tobacco Addiction Group). Where there are multiple eligible intervention arms within one study for a particular comparison and outcome, we do not combine these arms but include them in the analysis separately, splitting the comparator arm across intervention arms to prevent double counting. Statistical heterogeneity is assessed using the I^2^ statistic, where a value greater than 50% is deemed to indicate substantial heterogeneity. We plan to assess publication bias using funnel plots where syntheses include 10 or more studies, however this is not yet true for any of our analyses.

Table 1: Risk of bias (RoB) judgements

| **Study ID** | **Cochrane RoB 1 tool domain** | | | | | **Overall RoB judgement** | **Justification for overall judgements of ‘unclear’ or ‘high’** |
| --- | --- | --- | --- | --- | --- | --- | --- |
|  | **Selection bias** | **Performance bias** | **Detection bias** | **Attrition bias** | **Reporting bias** |  |  |
| Begh 2021 | Low | High | Low | Low | Low | High | Non-blinded and intensity of treatment unmatched between arms |
| Bullen 2013 | Low | Low | Low | Low | Low | Low |  |
| Caponnetto 2013 | Low | Low | Low | Low | Unclear | Unclear | Unclear if original intention was to combine two of the intervention groups in analyses and reporting |
| Cobb 2021 | Low | Low | Low | Low | Low | Low |  |
| Dawkins 2020 | High | High | Low | High | Low | High | Were unable to randomise despite intentions; non-blinded and intensity of treatment unmatched between arms; differential loss to follow-up between study arms |
| Eisenberg 2020 | Low | Low | Low | Low | Low | Low |  |
| Ely 2013 | High | n/a | n/a | Low | Unclear | High | Non-randomised study |
| Hajek 2019 | Low | Low | Low | Low | Low | Low |  |
| Halpern 2018 | Unclear | High | Low | High | Low | High | Randomisation methods not reported; non-blinded and intensity of treatment unmatched between arms |
| Holliday 2019 | Low | High | Low | Low | Low | High | Non-blinded and intensity of treatment unmatched between arms |
| Lee 2018 | Low | Low | Low | Low | Low | Low |  |
| Lucchiari 2020 | Low | Low | Low | Low | High | High | 12-month follow-up data not reported |
| Myers Smith 2021 | Low | Low | Low | Low | Low | Low |  |
| Polosa 2015 | High | n/a | n/a | Low | Unclear | High | No comparator; unable to determine pre-specified outcomes |
| Pulvers 2020 | Low | High | Low | Low | Low | High | Non-blinded and intensity of treatment unmatched between arms |
| Russell 2021 | Unclear | Low | Low | Low | Unclear | Unclear | Randomisation methods not reported; unable to determine pre-specified outcomes |

## Subgrouping of meta-analyses by flavour category (using random-effects meta-analyses)

### Nicotine EC versus NRT

#### Figure 1.1: Smoking cessation outcome


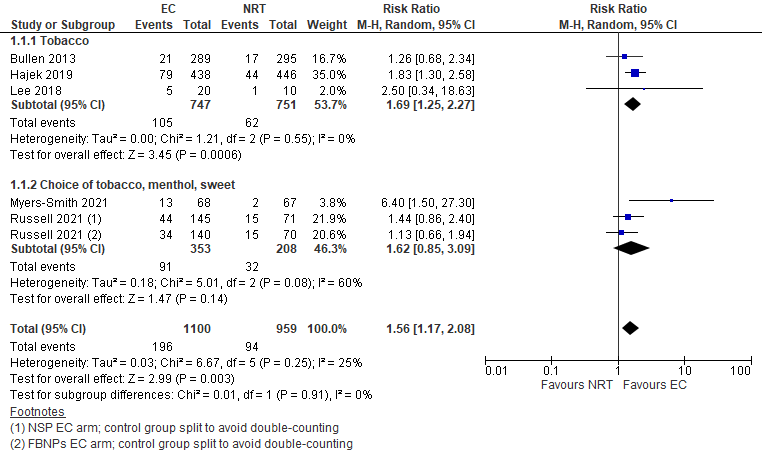


### Nicotine EC versus non-nicotine EC

#### Figure 2.1: Smoking cessation outcome


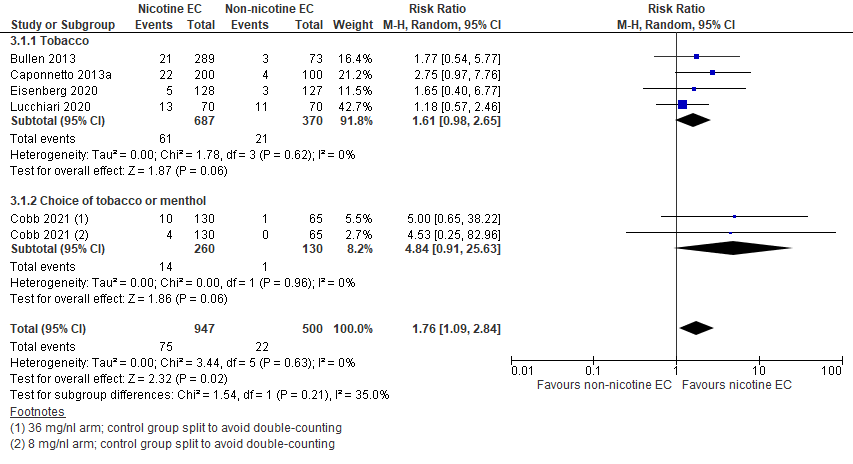


#### Figure 2.2: Long-term product use outcome


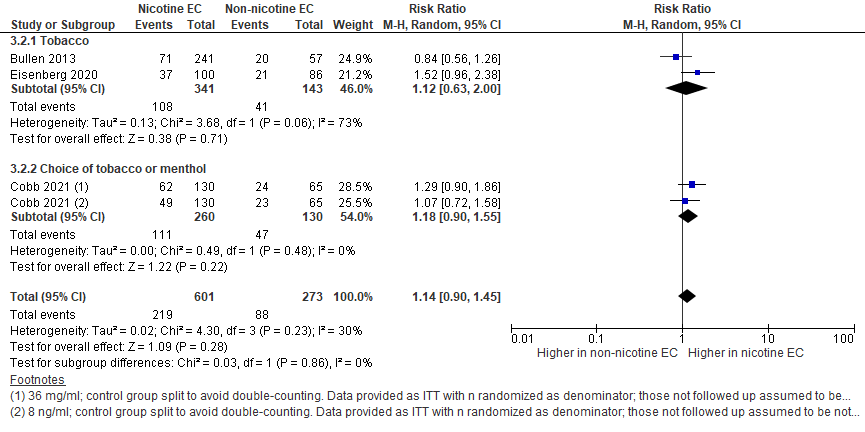


### Nicotine EC vs behavioural support only or no intervention

#### Figure 3.1: Smoking cessation outcome


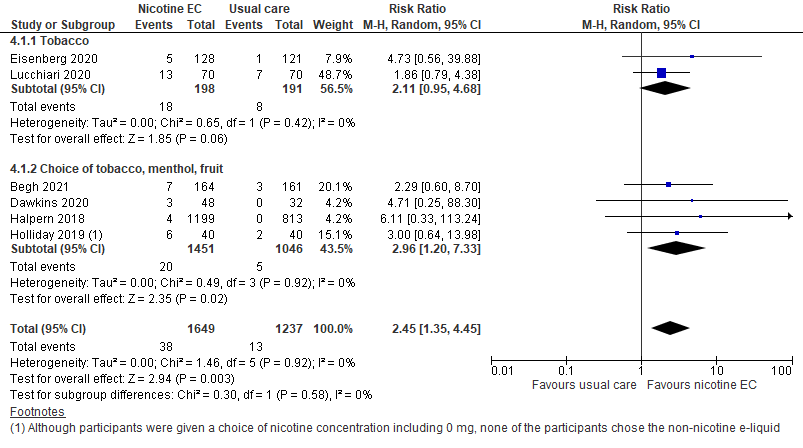


## *Table 2. Results from meta-analyses subgrouped by flavour (fixed-effects)*

| **Comparison** | **Outcome** | **Number of studies** | **I^2^ for subgroup differences** | **P value for subgroup differences** |
| --- | --- | --- | --- | --- |
| Nicotine EC v NRT | Smoking cessation | 3 tobacco only; 2 choice of tobacco, menthol or sweet | 0 | 0.68 |
|  | Study product use (Figure 1) |  | 98.3 | 0.00001 |
| Nicotine EC v non-nicotine EC | Smoking cessation | 4 tobacco only; 1 choice of tobacco or menthol | 27.9 | 0.24 |
|  | Study product use | 2 tobacco only; 1 choice of tobacco or menthol | 0 | 0.79 |
| Nicotine EC v behavioural support only or no support | Smoking cessation | 2 tobacco only; 4 choice of tobacco, menthol or fruit | 0 | 0.58 |

## Subgrouping of meta-analyses by flavour category (using fixed-effects meta-analyses)

### Nicotine EC versus NRT

#### Figure 4.1: Smoking cessation outcome


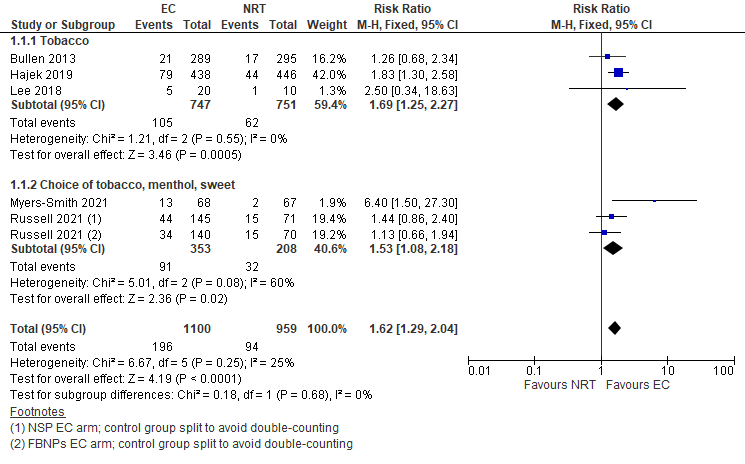


*Figure 4.2: Long-term product use outcome*


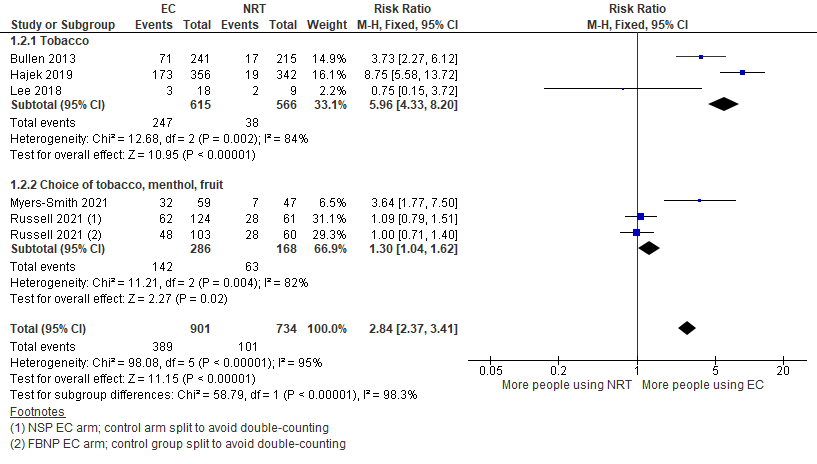


### Nicotine EC versus non-nicotine EC

#### Figure 5.1: Smoking cessation outcome


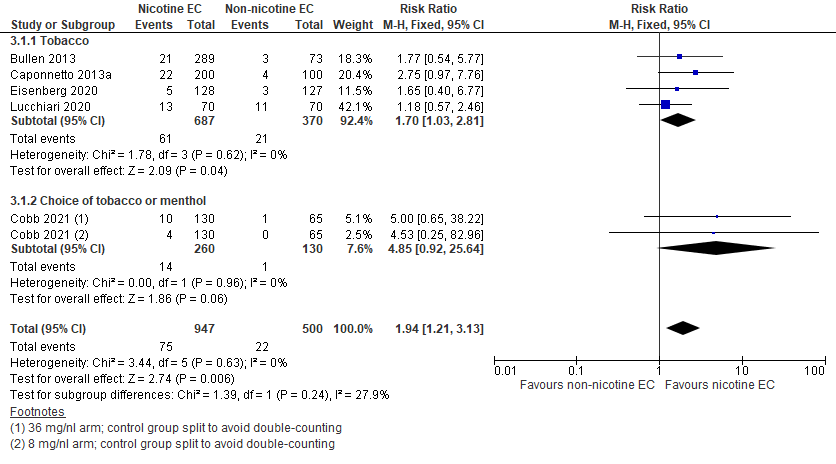


#### Figure 5.2: Long-term product use outcome


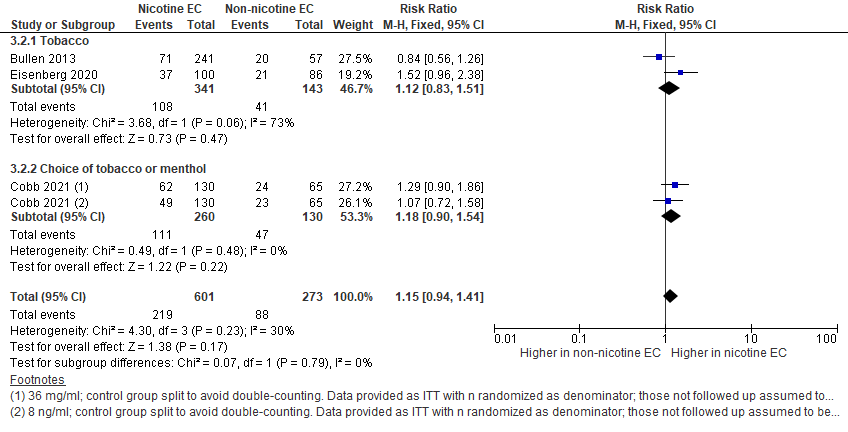


### Nicotine EC vs behavioural support only or no intervention

#### Figure 6.1: Smoking cessation outcome


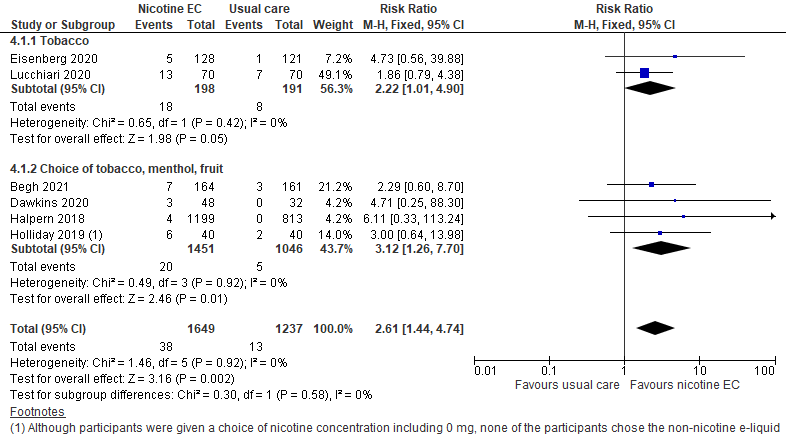


1. Hartmann-Boyce J, McRobbie H, Butler AR, Lindson N, Bullen C, Begh R, Theodoulou A, Notley C, Rigotti NA, Turner T, Fanshawe TR, Hajek P. Electronic cigarettes for smoking cessation. Cochrane Database of Systematic Reviews 2021, Issue 9. Art. No.: CD010216. DOI: 10.1002/14651858.CD010216.pub6 [↑](#footnote-ref-1)
